# Pediatric coronary artery fistula: echocardiographic case reports and literature review of treatment strategy

**DOI:** 10.1186/s40064-016-3276-0

**Published:** 2016-09-15

**Authors:** Guang Song, Jing Zhang, Weidong Ren, Ying Li, Ke Zhou

**Affiliations:** 1 Department of Ultrasound, Shengjing Hospital of China Medical University, 36# Sanhao Street, Shenyang, 110004 China; 2 Department of Cardiac Surgery, Shengjing Hospital of China Medical University, Shenyang, China

**Keywords:** Pediatric, Coronary artery fistula, Echocardiography

## Abstract

**Background:**

Coronary artery fistula (CAF) is a rare cardiac anomaly. Application of transthoracic echocardiography (TTE) is not fully illustrated in pediatric period. Meanwhile, the treatment strategy of CAF is still a controversial issue.

**Case presentation:**

Five cases of CAF with different types were presented. We also retrospectively reviewed 32 records of CAF in our institution from May 2001 to January 2015, including cardiac murmurs, symptoms, TTE diagnoses, complications, other anomalies, treatment and outcome. We summarized the most acceptable treatment strategy for pediatric patients. 71.9 % of all (23/32) had murmurs, 15.6 % of all (5/32) had symptoms. 24 patients received surgery or other imaging examination after TTE. 87.5 % of all (21/24) were correctly and accurately diagnosed by echocardiography, including pointing out the origin and outlet of CAF and complication. During the followup of all 32 patients, there was no spontaneous closure, heart failure, infective endocarditis or death case.

**Conclusions:**

TTE is a useful method that should be considered in the investigation and follow up of pediatric coronary artery fistula. The treatment strategy for pediatric patients should be individuation.

## Background

Coronary artery fistula (CAF) is a rare cardiac anomaly with incidence of 0.002 % in the general population (Fernandes et al. 1992). CAF comprises 0.31 % of all congenital heart defects (Caldwell and Ensing 1989). Complications secondary to CAF increase with age, including congestive heart failure (CHF), aneurismal dilatation of the coronary artery, infective endocarditis even death. Surgical risk and postoperative complications increase in patients over 20 years of age (Liberthson et al. 1979). So diagnosis as early as possible is good for patients. Transthoracic echocardiography (TTE) has been proved useful in adults and is sufficient for the diagnosis of CAF (Xie et al. 2014). Until now, the treatment strategy of CAF is still a controversial issue (particularly in asymptomatic patients). Herein, we present five different cases of CAF and summarize the acceptable treatment strategy.

## Case presentation

### Case 1

**Fig. 1:**
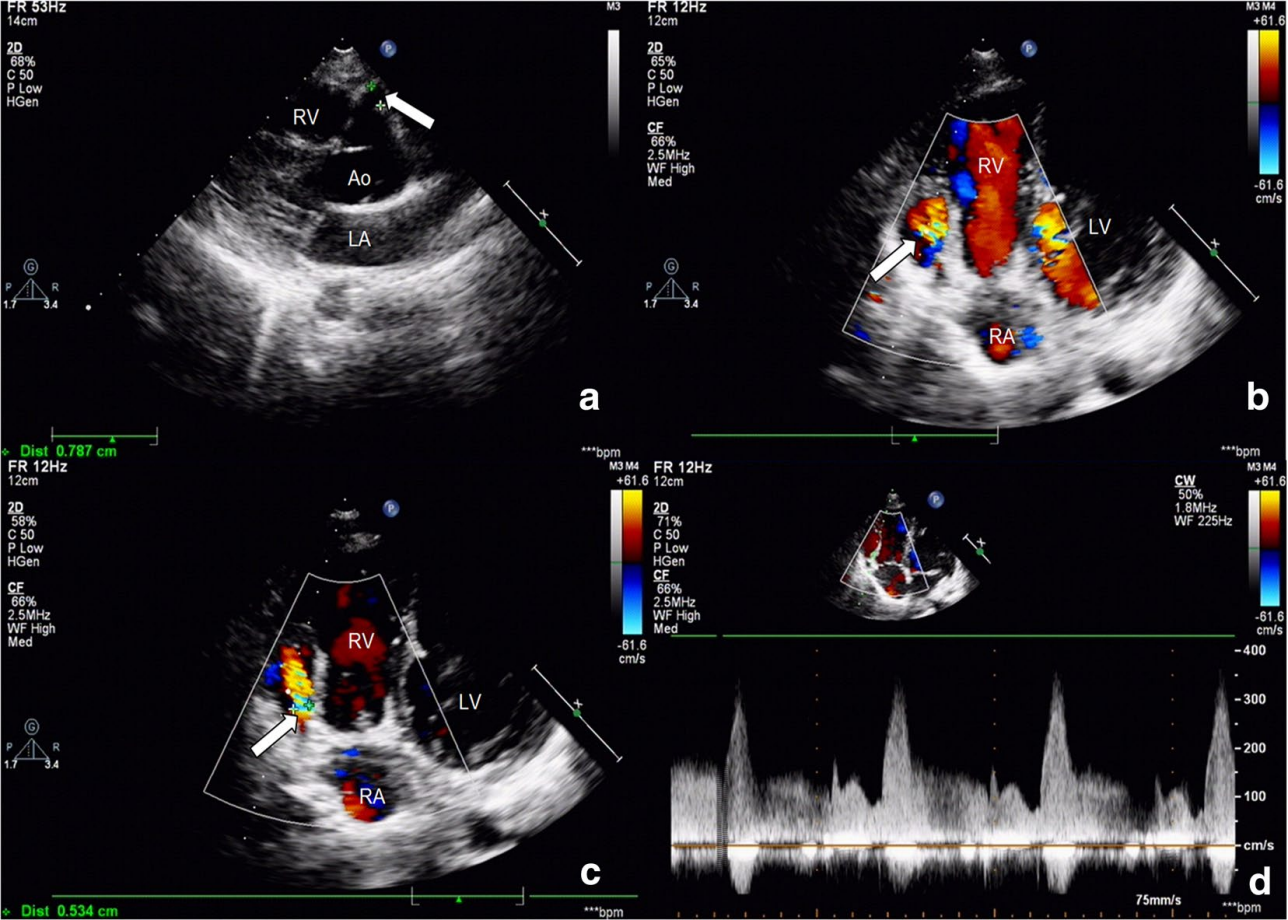
Right coronary artery-right ventricle (RV) fistula. **a** *Arrow* showed the dilated right coronary artery. **b** *Arrow* showed the aneurismal dilatation of right coronary artery. **c** *Arrow* showed fistula ostium. **d** The spectrum of fistula was continuous. *Ao* aorta, *LA* left atrium, *LV* left ventricle, *RA* right atrium, *RV* right ventricle

A 14-year-old boy was admitted to our hospital because of being heard cardiac murmur during school physical examination. He didn’t have any symptom in normal times. TTE revealed that the right coronary artery (RCA), of which diameter was significantly increased (7.9 mm), coursed to right about 25 mm (Fig. 1a, arrow). Then an aneurismal dilatation of RCA could be seen with a diameter of 27 mm. In this aneurysm, blood flow like a whirlpool (Fig. 1b, arrow). The blood flow through a fistula ostium, which was on the aneurysm wall, into the right ventricle (Fig. 1c, arrow). The peak velocity of blood flow is 3.5 m/s in the diastole (Fig. 1d). The surgery was performed 3 days later. The patient recovered well after surgery.

### Case 2

**Fig. 2:**
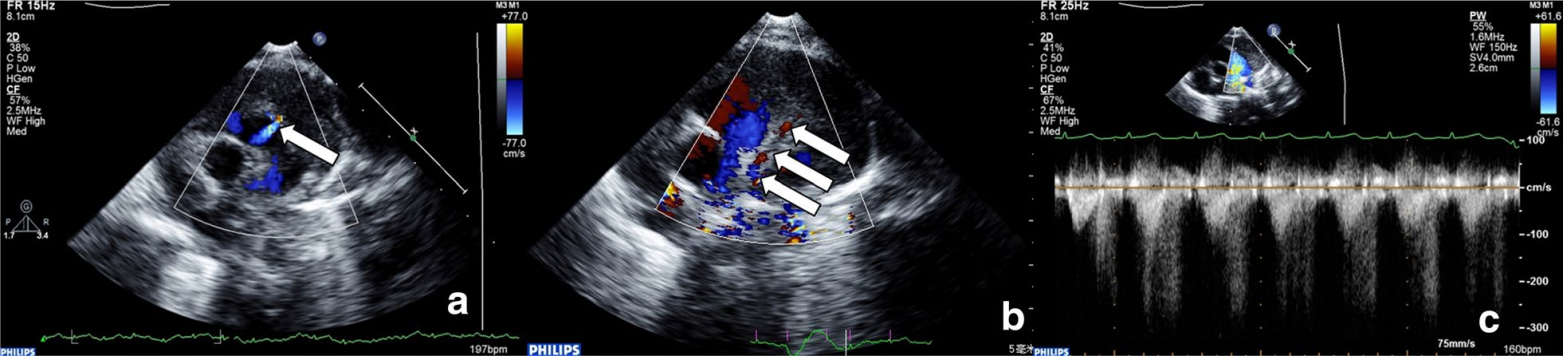
Left anterior descending-pulmonary artery fistula. **a** *Arrow* showed the abnormal blood flow in the pulmonary artery. **b** *Arrow* showed the origin of the fistula is left anterior descending. **c** The spectrum of fistula showed only in diastole

A 5-year-old girl came to our hospital for medical examination before primary school. She usually had no symptom. Cardiac auscultation could hardly hear any murmur. The electrocardiogram was normal. TTE showed that a diastolic high speed blood flow (3.0 m/s) was found on lateral wall of main pulmonary artery above the pulmonary valve annulus 12 mm (Fig. 2a, arrow). The abnormal blood flow pointed to the medial wall of main pulmonary artery. Reverse tracing of the abnormal blood flow, we found that the source is the left anterior descending (LAD) of left coronary artery (LCA) (Fig. 2b, arrow). The peak of the blood flow was more than 2.0 m/s in the diastole (Fig. 2c). After 3 years’ follow-up, she was still health.

### Case 3

**Fig. 3:**
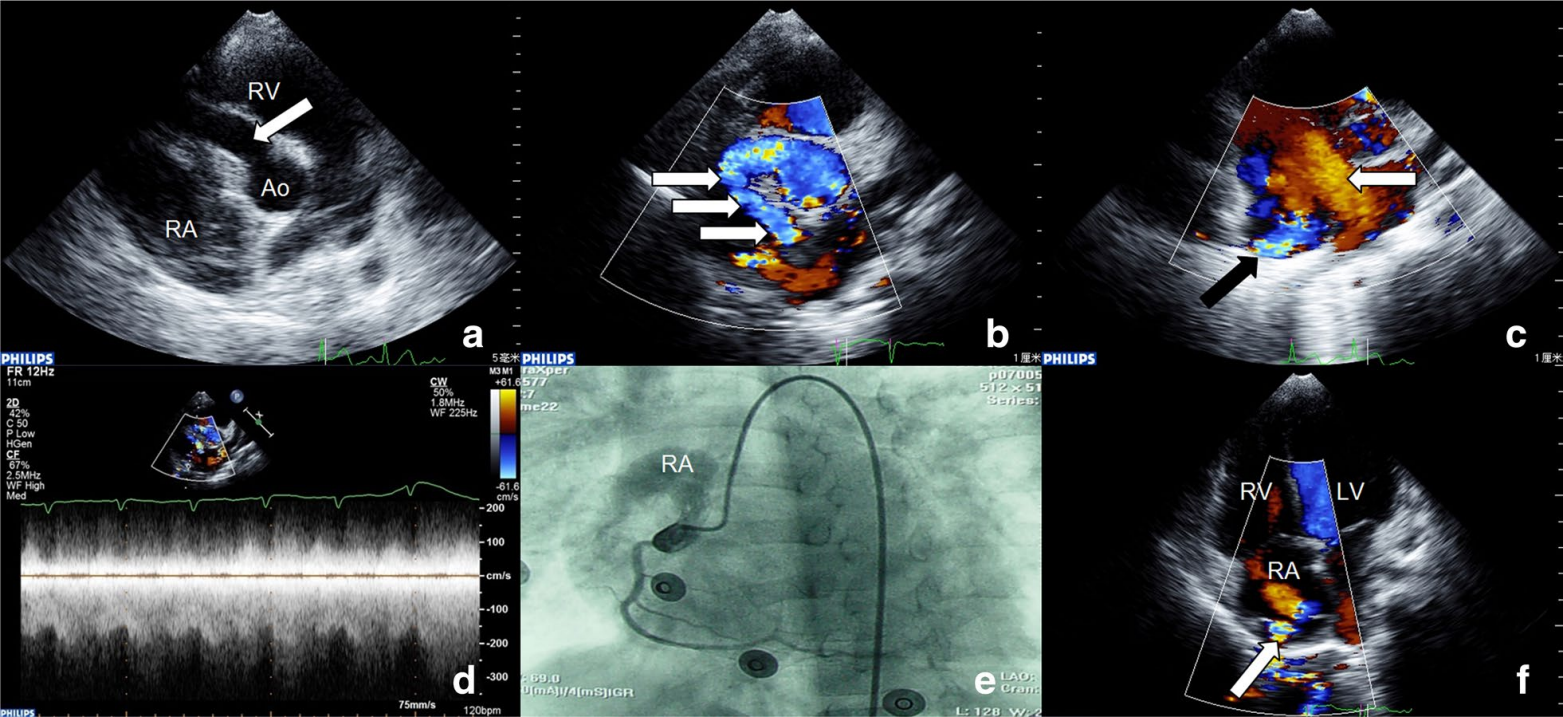
Right coronary artery-right atrium (RA) fistula. **a** *Arrow* showed the dilated right coronary artery. **b** *Arrow* showed the course of fistula. **c** *White arrow* showed fistula ostium, *black arrow* showed the atrial septal defect. **d** The spectrum of fistula is continuous. **e** Angiography confirmed the diagnosis before surgery. **f** *Arrow* showed a tiny residual shunting after surgery. *A*o aorta, *LV* left ventricle, *RA* right atrium, *RV* right ventricle

A 4-year-old girl came to our hospital because of being heard a grade 3/6 continuous murmur at the left side of the chest. She didn’t have obvious symptoms. TTE revealed that the diameter of RCA was significantly increased (9.0 mm) (Fig. 3a, arrow). Track the blood flow, we found that the coronary artery opened at the right atrium and the spectrum of fistula was continuous (Fig. 2b–d). At the same time, mild pulmonary stenosis and a big atrial septal defect also have been detected (Fig. 2c, arrow). Angiography confirmed the diagnosis (Fig. 2e). The surgery was performed a week later. One week after surgery, another TTE was performed and found that a residual flow on the top of right atrium (Fig. 2f, arrow). Due to the small shunt volume, the doctor decided to take conservation treatment and follow-up. The patient was discharged with well recovery.

### Case 4

**Fig. 4:**
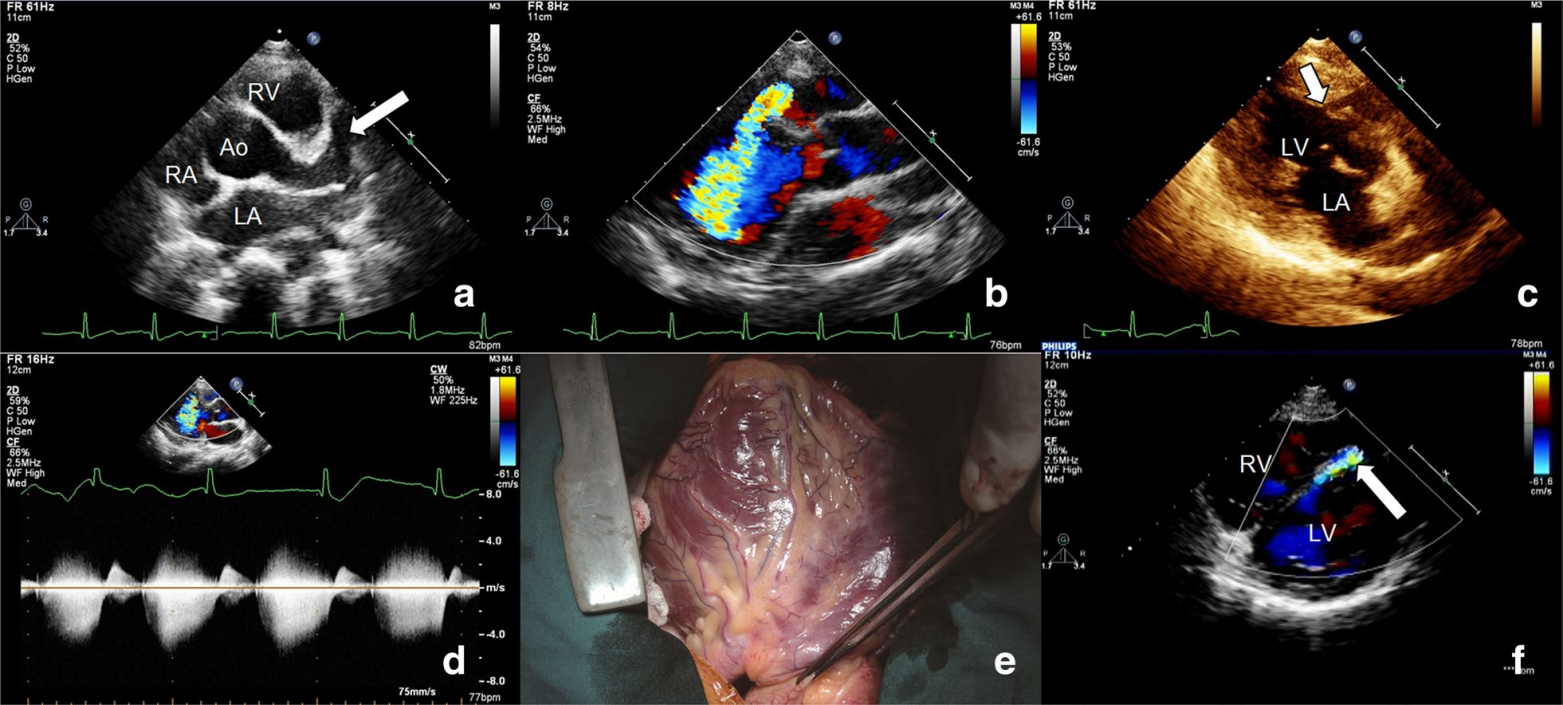
Left anterior descending-left ventricle (LV) fistula. **a** *Arrow* showed the dilated left anterior descending. **b** Color Doppler showed the course of fistula. **c** *Arrow* showed fistula ostium. **d** The spectrum of fistula showed only in diastole. **e** Surgery confirmed the diagnosis. f *Arrow* showed a tiny residual shunting after surgery. *Ao* aorta, *LA* left atrium, *LV* left ventricle, *RA* right atrium, *RV* right ventricle

A 10-year-old girl was admitted to hospital because of a diastolic cardiac murmur of grade 3/6~4/6 in the left side of the chest. TTE showed LAD was dilated (12 mm) (Fig. 4a, arrow). The LAD coursed to left from the posterior wall of the main pulmonary artery, then around to the front of pulmonary artery. The fistula ostium was on the middle part of ventricular septum (Fig. 4c), and its size was 5 mm. The spectrum of fistula revealed the shunt mainly occurred in diastole and peak speed was more than 4.0 m/s (Fig. 4d). Intraoperative findings confirmed the TTE diagnosis (Fig. 4e). One month after surgery, another TTE was performed and found there was a residual flow (Fig. 4f, arrow). The doctor decided not to carry out the second operation. The patient was healthy with follow-up until now.

### Case 5

**Fig. 5:**
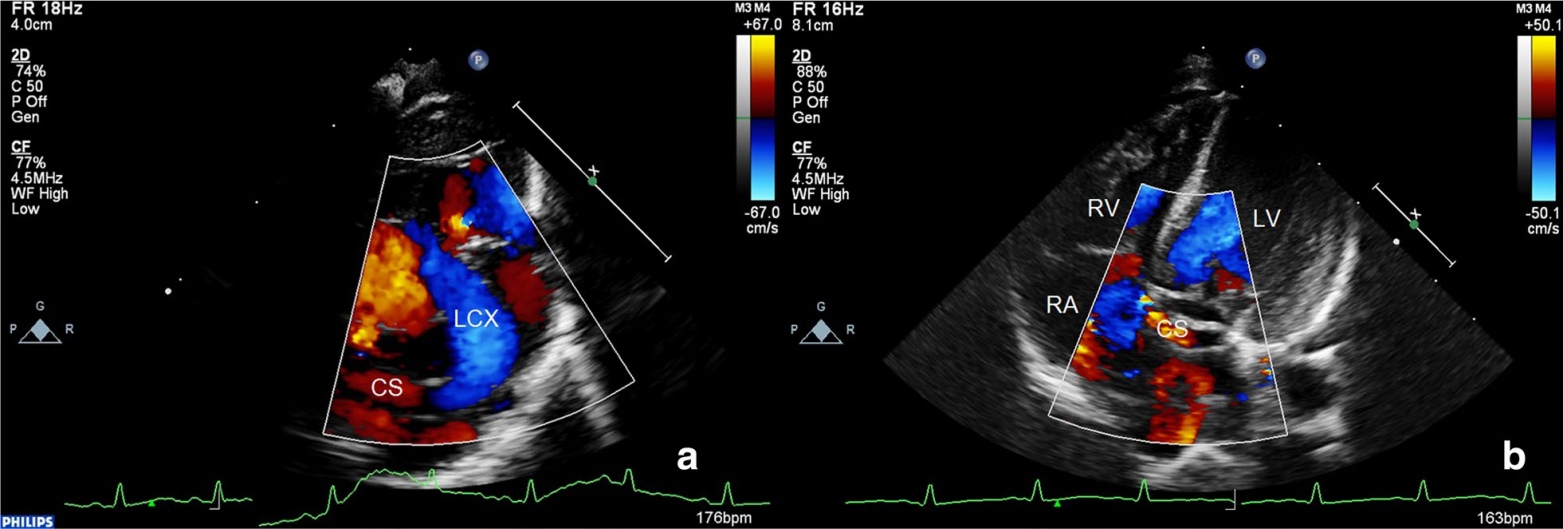
Left circumflex (LCX)-coronary sinus (CS) fistula. **a** LCX was dilated and connected with CS. **b** CS was connected with right atrium (RA). *CS* coronary sinus, *LCX* left circumflex, *LV* left ventricle, *RA* right atrium, *RV* right ventricle

A 13-day-old male premature infant was admitted to our hospital for medical treatment because of progressive dyspnea. Cardiac auscultation revealed a grade 3/6 continuous murmur at the left side of the chest. TTE showed coronary artery fistula from the left circumflex to coronary sinus (Fig. 5). Computed tomogram angiography confirmed the diagnosis. The doctor decided to take the conservation treatment for the patient. Two weeks later, the patient was discharged with increasingly improving physical condition. The patient is 2.5 years of age now, and is asymptomatic with a normal exercise capacity.

## Discussion

We retrospectively reviewed the medical records of our institution (Department of Ultrasound at the Shengjing Hospital of China Medical University) from May 2001 to January 2015 to search for children with CAF (Table 1).

**Table 1:** Clinical data, TTE and outcome

	Age	Gender	Symptoms	TTE			Other imaging	Treatment
				Origin	Outlet	Other		
1	5Y	M	Murmur	RCA	RV	None		Surgery
2	2Y	F	Murmur	RCA	RV	None	CTA	Surgery
3	7Y	M	Fatigue	RCA	RV	Aneurysmal dilatation		Surgery
4	14Y	M	Murmur	RCA	RV	Aneurysmal dilatation		Surgery
5	1Y	F	Murmur	LAD	RV	None		Surgery
6	3Y	M	Murmur	RCA	RV	None		Surgery
7	16Y	M	None	LAD	RV	None		Surgery
8	2Y	F	Murmur	RCA	RV	PS		Surgery
9	2Y	M	None	RCA	RV	None		Surgery
10	8Y	F	Murmur	RCA	RV	None		Surgery
11	5Y	F	Murmur	RCA	RV	VSD		Surgery
12	10Y	F	Murmur	RCA	RV	None		Transcatheter closure
13	52d	F	Murmur	RCA	RV	PS, CHF		Conservation
14	44d	F	Murmur	RCA	RV	None		Conservation
15	60d	M	None	LCA	RV	None		Conservation
16	1d	M	None	RCA	RV	None		Conservation
17	2d	F	Murmur dyspnea	LCA	RV	PAH		Conservation
18	5Y	F	Murmur	RCA LAD	PA	Mitral valve prolapse		Surgery
19	11Y	F	None	Unknown	PA	None	RCA by angiography	Conservation
20	5Y	M	None	Unknown	PA	None	RCA by angiography	Conservation
21	6Y	M	Dyspnea	Unknown	PA	None	LCX by angiography	Conservation
22	6Y	M	Murmur	LAD	PA	ASD		Conservation
23	5 M	F	None	LAD	PA	None		Conservation
24	4Y	F	Murmur	LCA	RA	None	CTA	Surgery
25	1Y	F	Murmur	RCA	RA	CHF, PAH		Surgery
26	7Y	F	Murmur	RCA	RA	PE		Surgery
27	4Y	F	Murmur	RCA	RA	ASD,PS	Angiography	Surgery
28	7Y	F	Murmur	LCA	RA	None		Surgery
29	57d	F	Murmur	RCA	RA	PFO		Conservation
30	11Y	F	Murmur	LAD	LV	None	CTA	Surgery
31	1Y	F	Dyspnea murmur	LCX	LV	VSD, PDA, PAH		Surgery
32	13d	M	Dyspnea murmur	LCX	CS	CS stenosis	CTA	Conservation

*RCA* right coronary artery, *LCA* left coronary artery, *LCX* left circumflex, *LAD* left anterior descending, *RV* right ventricle, *PA* pulmonary artery, *RA* right atrium, *LV* left ventricle, *CS* coronary sinus, *PS* pulmonary stenosis, *CHF* congestive heart failure, *ASD* atrial septal defect, *PAH* pulmonary arterial hypertension, *PFO* patent foramen ovale, *PE* pericardial effusion, *VSD* ventricular septal defect, *PDA* patent ductus arteriosus

71.9 % of all (23/32) had murmurs, 15.6 % of all (5/32) had symptoms. CAF were diagnosed in 32 patients. 56.3 % of all (18/32) had isolated fistula, while the rest 14 patients associated with other cardiac anomalies. Fistula originating from the right coronary artery (RCA) accounted for 59.4 %, with 37.5 % from the left coronary artery (LCA), 3.1 % from both coronary arteries. The fistula might drain into the right ventricle (53.1 %), the pulmonary artery (18.8 %), the right atrium (18.8 %), the left ventricle (6.3 %) or the CS (3.1 %). 24 patients received surgery or other imaging examination after TTE, including 19 cases with surgery, 1 case with transcatheter treatment, 4 cases with angiography and 4 cases with computed tomogram angiography. 87.5 % of all (21/24) were correctly and accurately diagnosed by echocardiography, including pointing out the origin and outlet of CAF and complication. 3 cases (Nos. 15, 16, 17) could not show the origins of CAF, which are the tiny branches of coronary artery diagnosed by angiography. 12 patients received conservation treatment. During the follow-up of all 32 patients, there was no spontaneous closure, heart failure, infective endocarditis or death case.

The cardiac murmur, the dilated coronary artery and abnormal blood flow could lead the sonographer to suspect the existence of CAF. Although limited with the size of chambers and vessels, CAF was still easily detected by TTE due to pediatric good imaging quality.

The complications of CAF include: (1) aneurysmal dilatation of the coronary artery: aneurysmal dilatation was found in 19–26 % of CAFs (Said and Gamal 1995; Urrutia et al. 1983). (2) Congestive heart failure: some research revealed that congestive heart failure is reported in 6 % of patients with CAF under age 20 years (Starc et al. 1986). The pulmonary vascular resistance increased in neonatal period and decreased at between 2 and 3 months of age. So congestive heart failure caused by CAF usually presented at between 2 and 3 months of age. Congestive heart failure in neonatal period is rare. The termination of the fistula into low-pressure cardiac structures such as the right atrium and coronary sinus is also likely to result in a larger left-to-right shunting than if the fistula terminated in high-pressure structures like the left ventricle (Li et al. 2011). (3) infective endocarditis: the incidence of infective endocarditis with patients under 20 years old is 3 %. Meanwhile, the fistula usually was medium or large (Liberthson et al. 1979). Due to the very early diagnosis, all patients in our study got the diagnosis before they have serious symptoms or complications. So the very early diagnosis is good for patients, especially for those over 20 years of age (Liberthson et al. 1979).

Current treatment options include conservation treatment, surgery and transcatheter closure. The management of CAF is still a controversial issue (particularly in asymptomatic patients).

Treatment strategy for asymptomatic CAF: (1) small/low-flow shunting fistula do not require a specific procedure for closure simply (like our case 2). However, closure of fistula may be recommended: (1) if the patient is considered to be at high risk for later infective endocarditis; (2) if longitudinal follow-up is not feasible; (3) if the patient is undergoing an invasive procedure for some other cardiac problem (Latson 2007). (2) Big/high-flow shunting: patients need surgery or transcatheter closure although fistula may not produce symptoms during infancy or childhood. There is a risk that heart failure may develop owing to chronic left ventricular volume overload later in life (Mavroudis et al. 1997). Also, the incidence of death increase significantly after two decades (Hong et al. 2004).

Treatment strategy for symptomatic CAF: (1) neonate/infant: symptomatic CAFs have been associated with significant morbidity and mortality. Surgery can be performed with comparatively low risk and is essentially curative. The symptoms from a large left-to-right shunting are often controllable by typical medical management. Eventually 50 % of fistulas become asymptomatic due to decrease in relative size of the fistula and shunting with growth, or spontaneous regression (Hsieh et al. 2002). Those who do not respond to medical management need intervention. (2) elder: elective closure of CAF either by surgery or by transcatheter techniques should be taken into consideration.

The first successful surgical was operated by Bjork and Crafoord in 1947, and the first therapeutic embolization was performed by Zuberbuhler et al. in 1974. Previous study showed that a very low morbidity and mortality rate (0–4 %) when fistulas are operated on in infancy and childhood (Schumacher et al. 1997). Although repair of coronary artery fistula is relatively mature, postoperative complications may happen occasionally, including thrombosis, angina with coronary thrombosis, myocardial infarction and rupture of aneurysm. The incidence of each is less than 2 % (Mangukia 2012). There are also 20–30 % of patients with residual shunting using coronary angiography after CAF repair. Meanwhile, 10 % had demonstrable recurrence of fistula without hemodynamic disturbance (Cheung et al. 2001; Valente et al. 2010). Although postoperative coronary angiography should be done, some parents refused to perform it because of invasion and radiation. So we use TTE to evaluate the effect of surgery and to follow up just like Case 3.

In conclusion, TTE is a useful method that should be considered in the investigation and follow up of pediatric coronary artery fistula. The treatment strategy for pediatric patients should be individuation.

## Consent

This study was approved by the Regional Ethics Committee of our hospital and that permission was granted to all the authors to access the patient’s data. Written informed consents were obtained from legal parents of all patients for the publication of this report and any accompanying images.

## Abbreviations

CAF: coronary artery fistula
TTE: transthoracic echocardiography
